# Distinct chromosomal mutation associated with cefiderocol resistance in *Acinetobacter baumannii*: a combined bioinformatics and mass spectrometry approach to unveil and validate the *in vivo*-acquired chemoresistance

**DOI:** 10.3389/fmicb.2024.1480322

**Published:** 2024-12-18

**Authors:** Lavinia Morosi, Davide Golzato, Linda Bussini, Hygerda Guma, Federica Tordato, Federica Armanini, Zian Asif, Francesco Carella, Paola Morelli, Michele Bartoletti, Giorgio Da Rin, Erminia Casari, Giuseppe Martano, Maria Rescigno, Nicola Segata, Sara Carloni, Valeria Cento

**Affiliations:** ^1^IRCCS Humanitas Research Hospital, Rozzano, Italy; ^2^Department CIBIO, University of Trento, Trento, Italy; ^3^Infectious Diseases, IRCCS Humanitas Research Hospital, Milan, Italy; ^4^Department of Biomedical Sciences, Humanitas University, Milan, Italy; ^5^Microbiology and Virology, IRCCS Humanitas Research Hospital, Milan, Italy; ^6^Institute of Neuroscience, National Research Council of Italy (CNR) c/o Humanitas Mirasole S.p.A, Rozzano, Italy

**Keywords:** *Acinetobacter*, cefiderocol, CRAB, variant calling analysis, LC–MS/MS

## Abstract

*Acinetobacter baumannii* is a significant public health concern due to the emergence of antibiotic-resistant strains. Cefiderocol (FDC), a novel siderophore cephalosporin, has shown promise as a last-line treatment for multidrug-resistant Gram-negative bacteria. However, the emergence of *in vivo*-acquired FDC-resistant *A. baumannii* strains highlights the need for advanced tools to identify resistance-associated genomic mutations and address the challenges of FDC susceptibility testing. This study aims to characterize a novel mutation responsible for FDC resistance in *A. baumannii* and to develop a workflow that integrates genomic and functional analyses for improved antimicrobial resistance monitoring. We examined two carbapenem-resistant *A. baumannii* isolates from bacteremia cases in two patients (*A. baumannii*_5406 from patient A and *A. baumannii*_5577 from patient B). Initial whole-genome sequence BLAST typing identified both as the same strain. However, a minimum inhibitory concentration (MIC) analysis showed that *A. baumannii*_5406 was resistant to FDC, while _5577 was not. Further variant calling analysis revealed a novel chromosomal mutation in a gene encoding a TonB-dependent receptor homolog, which is involved in ferric-siderophore and heme uptake. This mutation causes a premature stop codon, likely impairing the receptor’s function. Mass spectrometry confirmed that the FDC-resistant strain exhibited reduced antibiotic uptake and intracellular accumulation. This study demonstrates the utility of combining genomic and functional analyses to detect emerging mutations associated with antibiotic resistance. The variant calling approach, together with LC–MS/MS technology, offers a valuable complement to traditional susceptibility testing in clinical settings, potentially improving the identification and monitoring of FDC resistance in *A. baumannii*. Additionally, this workflow could aid in the epidemiological tracking of resistant strains.

## Graphical abstract

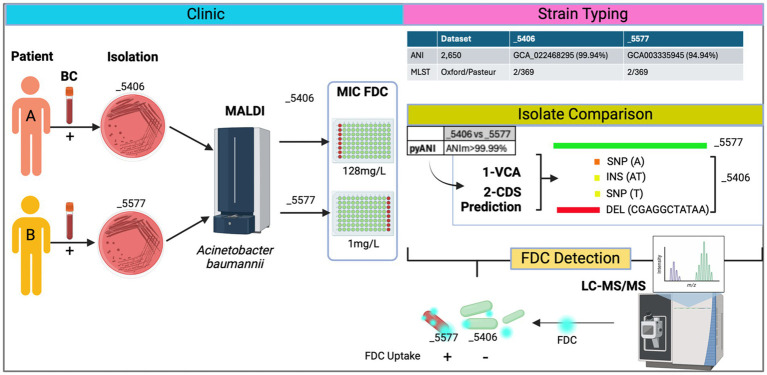

## Introduction

In recent years, the Gram-negative bacterium *Acinetobacter baumannii* has gained public notoriety for its ability to cause a wide range of nosocomial infections and to acquire different mechanisms of resistance (Dijkshoorn et al., 2007; Ibrahim et al., 2021). This opportunistic pathogen is associated with mortality rates up to 80%, especially in patients with multidrug-resistant bacteremia and septic shock (Busani et al., 2017; Russo et al., 2019). In 2017, the World Health Organization (WHO) identified the carbapenem-resistant *A. baumannii* (CRAB) as a major public health concern and critical priority pathogen, thus highlighting the urgent need for new therapeutic options (Tacconelli et al., 2018; WHO, 2023).

Cefiderocol (FDC; S-649266) is a novel siderophore cephalosporin that was recently approved for the treatment of adults with complicated Gram-negative urinary tract infections (cUTIs), hospital-acquired pneumonia (HAP), and ventilator-associated pneumonia (VAP), including infections caused by CRAB (Padovani et al., 2023; Ito et al., 2016; Syed, 2021).^1^ A broad variety of Gram-negative bacteria, including Enterobacterales (such as *Escherichia coli, Klebsiella pneumonia, Citrobacter freundii, Enterobacter cloacae, and Serratia marcescens*), non-fermenters (such as *Pseudomonas aeruginosa*, *A. baumannii*, and *Burkholderia cepacia*), and Proteaceae (including *Proteus mirabilis, Proteus vulgaris*), are susceptible to the effect of FDC (Syed, 2021; Dobias et al., 2017). This new cephalosporin demonstrates remarkable stability against a wide range of serine-type and metallo-type carbapenemases, as well as extended-spectrum *β*-lactamases (ESBLs) (El-Lababidi and Rizk, 2020). FDC mimics the activity of siderophores produced by Gram-negative bacteria under iron stress conditions, featuring a terminal chlorocatechol group in the C-3 side chain. This group has the capability to chelate ferric iron and penetrate the outer membrane of Gram-negative bacteria, gaining access to the periplasmic space through the iron entrance system (Soriano and Mensa, 2022). This mechanism, referred to as “self-promoted uptake” or “Trojan horse,” presents a novel approach to overcoming bacterial resistance mechanisms (Noinaj et al., 2010). The transfer of FDC from the outer membrane to the periplasmic space occurs through either passive diffusion via porin channels or active transport facilitated by TonB-dependent siderophore receptors (TBDRs) including PiuA and PirA. TBDRs represent a pivotal class of outer membrane proteins that play a fundamental role in nutrient acquisition, particularly within the host’s iron-restricted environments, and have garnered significant attention due to their association with antibiotic resistance in *A. baumannii* and *P. aeruginosa* (Padovani et al., 2023; Teran et al., 2024; Egge et al., 2024; Moynié et al., 2017). Indeed, the TonB protein, along with its associated inner membrane proteins ExbB and ExbD, energizes TBDRs, allowing them to actively transport iron from the extracellular environment into the bacterial periplasm (Noinaj et al., 2010). FDC can use that system to reach the periplasmic space, where it binds to penicillin-binding proteins (PBPs), mainly PBP3, thus impairing peptidoglycan synthesis and causing cell death (Syed, 2021; Dobias et al., 2017; Malik et al., 2020; Ong’uti et al., 2022; McElheny et al., 2021; Nurjadi et al., 2022). Recent *in vitro* investigations have unveiled that mutations in PBPs and the catecholate siderophore receptor (*CirA*), driven by the selective pressure of β-lactamase (NDM), mediate FDC resistance in Enterobacterales such as *K. pneumoniae* and *E. cloacae*, as well as in *A. baumannii* and *P. aeruginosa* (*REFs*) (Malik et al., 2020; McElheny et al., 2021; Nurjadi et al., 2022).

The gold standard for *in vitro* FDC susceptibility testing is broth microdilution (BMD) in iron-depleted cation-adjusted Mueller–Hinton Broth (ID-CAMHB), as recommended by CLSI and EUCAST (European Committee on Antimicrobial Susceptibility Testing, n.d.; Clinical and Laboratory Standards Institute, 2024; Hackel et al., 2019; Simner and Patel, 2021). Although various commercial systems for FDC susceptibility testing have been introduced, they have faced significant accuracy issues, prompting a recent warning from EUCAST.^2^ While disk diffusion can be useful for preliminary screening, its broad technical uncertainty often necessitates retesting many isolates using broth microdilutions to achieve definitive results on MIC (Bonnin et al., 2022; Morris et al., 2021).

One complicating factor in translating *in vitro* research to *in vivo* efficacy is the variation in iron availability, which is reflected in the differing regulation of iron transport mechanisms including TonB-dependent transporters and receptors (Page, 2019; Runci et al., 2019).

This study seeks to develop an integrated research framework that combines genomic and functional analyses, facilitating the identification of clinically significant genetic markers to enhance the monitoring of antimicrobial resistance.

In particular, the variant calling analysis allows the identification of 10 bp mutation into a novel CDS homolog of TonB-dependent receptor. Clinical application of the variant calling analysis provides swift and accurate profiling of the patient’s isolate. Furthermore, LC–MS/MS technology allows rapid detection of FDC within pathogens, enabling an *in vitro* functional assay of the predominant FDC-resistant mechanisms. Introducing the variant calling analysis and the MS-based FDC detection into clinical practice could enhance the monitoring of *in vivo* resistant *A. baumannii* isolates.

## Materials and methods

### Bacterial strains and phenotypic drug-resistance testing

The FDC-resistant *A. baumannii_5406* strain was isolated in 2022 from blood cultures (BCs) of patient A on day 24 during a small hospital outbreak. An FDC-susceptible *A. baumannii_5577*, isolated from BC of patient B from the same outbreak, was used as a reference. Bacterial identification was performed through MALDI-ToF (Bruker Diagnostics). Antimicrobial susceptibility testing was carried out using the M50 Automated Microbiology System (Becton Dickinson Diagnostic Systems, Sparks, MD, USA) with NMIC-474 panels. Results were interpreted according to the European Committee on Antimicrobial Susceptibility Testing (EUCAST) breakpoints 2023 (Breakpoint tables for interpretation of MICs and zone diameters, Version 13.0, 2023).^3^ FDC resistance was assessed by broth microdilution using ComASP^®^ Cefiderocol 0.008–128 μg/mL (Liofilchem).

### Bacterial cultures and CFU measurement

To evaluate the correlation between optical density at 600 nm (OD600) and colony-forming units (CFUs), the isolates were cultured in Mueller–Hinton broth (MHB) at 37°C with shaking at 120 rpm, and the exponential growth phase was employed to determine the OD600/CFUs correlation after bacterial washing in PBS. An OD600 of 1 corresponds to 1.11 × 10^8 CFUs. The OD600 value of 0.18, used for subsequent FDC uptake analysis, corresponds to 2 × 10^7 CFU/mL. For analysis of FDC uptake, the purified *A. baumannii_5577* and *A. baumannii_5406* were inoculated overnight at 37°C in ID-CAMHB as described by Ito et al. (2016) and reported by the Clinical and Laboratory Standards Institute. In brief, 400 mL of medium was combined with 40 g of cation-binding resin Chelex 100 (BioRad) to remove cations, including iron, and incubated at room temperature for 1 h. The resulting mixture was filtered to remove particulate matter. Subsequent pH adjustment to 7.3 using hydrochloric acid was followed by another filtration step. Essential minerals (Ca2+ 25 mg/L, Mg2+ 10 mg/L) were added in specified concentrations. To avoid the activation of existing metal beta-lactamases, we exclude Zn2+. All steps were conducted under sterile conditions.

### Setting of FDC uptake experiment in *Acinetobacter baumannii_5577* and *Acinetobacter baumannii_5406* bacterial strains

For analysis of FDC uptake, the purified *A. baumannii_5577* and *A. baumannii_5406* were inoculated overnight at 37°C in the ID-CAMHB medium. In brief, the overnight culture was restarted in ID-CAMHB until the mid-exponential phase at an OD_600_ of 0.6. For the antibiotic uptake experiment, 2×10^7 cells were used and checked for their normalization as well as CFU on plating. Next, 10 μg/mL of FDC (HY-17628-10 mg) was simultaneously introduced to the tested isolates, and the samples were incubated at 37°C, at 120 rpm, for 5 and 20 min. Following incubation, spectrophotometric measurement of the samples was performed, and 1ml of the sample was withdrawn and subjected to centrifugation at 16,000 rcf for 1 min at 4°C, to obtain the supernatant. The resulting pellet was washed three times with water, with each wash involving centrifugation at 16,000 rcf for 1 min at 4°C. The spectrophotometric measurement of the sample allowed for cell quantification following the washing steps. Lysis of the cell pellets was achieved using incubation with 10 mg/mL of lysozyme (VWR 12650-88-3, ultra-pure) and 10 mM of EDTA, followed by 15 cycles of 30-s sonication bursts. The efficacy of the lysis process was confirmed by total plating of one replicate, ensuring complete cell lysis.

### FDC quantification by LC–MS/MS

FDC uptake was quantified using LC–MS/MS detection on a TSQ Altis™ Triple Quadrupole Mass Spectrometer (Thermo Scientific) operating in positive ion mode. The transitions monitoring were m/z 752.30 > 285.21 (quantitation) and m/z 752.30 > 214.11 (confirmation) for FDC and m/z 455.16 > 156.04 (quantitation) and m/z 455.16 > 323.12 (confirmation) for cefazolin as internal standard. The positive ion voltage was set to 4900 V, with sheath gas, auxiliary gas, and sweep gas flow rates set at 55, 4.8, and 0, respectively. These values were experimentally selected to optimize the ionization of FDC. The ion transfer tube temperature was 250°C, and the vaporizer temperature was 275°C. Chromatographic separation was achieved on a Gemini-C18 column (50 mm × 2.0 mm, 5 μm particle size; Phenomenex Inc., Torrance, CA, USA) at 35°C at 0.2 mL/min using H2O plus formic acid 0.1% (mobile phase A) and CH3OH plus formic acid 0.1% (mobile phase B) under gradient conditions as follows: 10–90% B (0–4.5 min), 90% B (4.5–5 min), 90–10% B (5–5.1 min), and 10% B (5.1–15 min). Lysed cell pellets were added with acetonitrile plus formic acid to precipitate protein. After centrifugation at 15000 rcf, the supernatant was evaporated to dryness under N_2_ flux. The extracts were reconstituted in 200μl of mobile phase B 10%, and 10 μL were injected into the HPLC system. The study samples were assayed together with a five-point calibration curve in untreated bacterial cell pellets at concentrations ranging from 0.1 to 50 ng/sample. The limit of quantification (LOQ) was 0.1 ng/sample. The results of FDC uptake were calculated as the absolute amount of FDC (in nanograms) within the cells.

### Shotgun sequencing and analysis

The two isolates were grown at 37°C in MHB, and the whole DNA was extracted using the Quick-DNA Fungal/Bacterial Miniprep Kit Zymo research (Cat. No.:D6005). Sequencing library preparation of genomic DNA extracted from the two isolates was prepared using the Illumina DNA prep and Tagmentation kit. Libraries were sequenced (150-bp paired-ends reads) on a NovaSeq6000 instrument with S4 flowcell reagents (Illumina) at the University of Trento (Italy), after a cleaning step using 0.6x Agencourt AMPure XP beads.

Raw reads were pre-processed using Trim Galore (parameters: --stringency 5 --length 75 --quality 20 --max_n 2 --trim-n)^4^, resulting in a total of 10,802,024 and 9,419,450 of high-quality paired-end (weighted mean Q values >35) for *Acinetobacte*r_5406 and _5577, respectively. Genome assembly was performed with SPAdes (3) ver. 3.15.2 (Prjibelski et al., 2020) (parameters: --careful -k 21,33,55,77,99,127). Contigs shorter than 1,000 nucleotides were filtered out from the final assemblies. Average nucleotide identity (ANI) between isolate assemblies and the 2,650 NCBI genomes annotated as *A. baumannii* was computed using FastANI (version 1.33) (Jain et al., 2018).

FCS-GX (Astashyn et al., 2024) was used to clean the assembled contigs from potential contamination from organisms different than *A. baumannii* (NCBI taxonomy ID: 470). Completeness and contamination of the resulting genome assemblies were assessed using CheckM v1.1.2 (Parks et al., 2015). Genomes of Acinetobacter_5406 (BioSample: SAMN41943660, genome accession: JBEUFA000000000) and Acinetobacter_5577 (BioSample: SAMN41943661, genome accession: JBEUEZ000000000) were deposited in the NCBI under BioProject PRJNA1126554. CDS prediction and annotation of *A. baumannii* genomes were performed using Prokka version 1.14 (executed with additional flags --force --addgenes --addmrna) (Seemann, 2014). Multi-locus sequence typing (MLST) of the genome assemblies was performed on PubMLST (Jolley et al., 2018). Genomic similarity between the assemblies of isolates _5406 and _5577 was assessed using pyANI (version 0.2.12) (Pritchard et al., 2015), which performs whole-genome alignment using MUMmer (Marçais et al., 2018). Variant detection was performed using the tool Snippy Version 4.6.0 (Seemann, 2014)^5^ with default parameters, choosing *A. baumanii*_5577 as the reference genome and *A. baumanii*_5406 reads as input. No changes were made to the filtering criteria used by Snippy to select significant variants. Functional annotations of the hypothetical protein affected by the variants detected by Snippy were obtained with blastp mapping against database nr/nt, directly from the web application of the NCBI (Camacho et al., 2009), following the guidance document for cluster analysis of whole-genome sequence data.^6^

The presence of TonB-dependent receptor gene homologs across other *A. baumannii* strains was assessed by mapping with blastn (version 2.13.0+), the putatively active gene from *A. baumannii_5577* on all the genomes of *A. baumannii* belonging to ST 2 and 369 (Oxford and Pasteur schemes) that were available for download on PubMLST (*n* = 5,264, at 6 June 2024). Homologous sequences were then extracted and aligned using multiple sequence alignment (MSA) tool muscle v 5.1 (Edgar, 2004).

The 10-base deletion was confirmed through PCR sequencing. The PCR fragment was generated using the primers FW_TGCCGTCTCGAATTGTTTGG and REV_CTATCAAGCATTGCCACGGG, with the sequencing carried out using the REV primer.

### Protein prediction

The putative codons from the DNA sequence were examined to ensure the correct translation and identify any potential functional domains or motifs that might be important for the protein’s activity. Protein sequences were analyzed using Phyre2 (Bonnin et al., 2022) to predict the structural characteristics of the hypothetical protein translated from the CDS with locus tag EECGJPIA_00294. Phyre2 employs advanced homology modeling techniques to infer the likely three-dimensional structure and functional aspects of the protein based on known protein structures. Following the Phyre2 analysis, PyMOL [Schrödinger and DeLano (2020) was utilized for detailed characterization of the predicted 3D structures].^7^ PyMOL’s sophisticated visualization capabilities enabled a thorough examination of various structural components, such as alpha helices and beta sheets. By integrating the predictions from Phyre2 with the structural visualizations in PyMOL, a comprehensive picture of the hypothetical protein’s architecture and possible biological activities was achieved.

## Results

### Case report

In 2022, a 67-year-old man (Patient A) presented to the Emergency Department with fever and dyspnea (D0). Upon admission, blood tests revealed hyperleukocytosis, anemia, and thrombocytopenia, raising suspicion for acute myeloid leukemia, which was later confirmed by a bone marrow biopsy. A lung CT scan documented multifocal bilateral pneumonia. Piperacillin/tazobactam and levofloxacin were promptly introduced, along with hydroxyurea and non-invasive ventilation. Blood cultures, serum *β*-D-glucan and galactomannan, urinary antigens, and multiplex RT-PCR for respiratory pathogens were negative. Fibro-bronchoscopy for bronchoalveolar lavage was not performed due to severe respiratory failure. In the following days, the patient achieved apyrexia and progressive de-escalation of respiratory support. On D13, upon rapid worsening of hyperleukocytosis, patient A started a chemotherapy regimen with 3-day daunorubicin, which was followed by 7-day cytarabine. Three days later, the patient developed febrile neutropenia, with negative blood cultures (BCs). Meropenem was initially introduced, but due to persistent fever and detection of urinary and rectal colonization by FDC-susceptible CRAB, it was replaced with FDC on D18. The initial isolate was not collected, making it impossible to incorporate it into subsequent analyses. On D24, BCs turned positive, and a FDC-resistant *Acinetobacter baumannii* was isolated (*A. baumannii_5406*) (Table 1). Antibiotic therapy was then shifted to colistin monotherapy. However, the clinical conditions of patient A rapidly deteriorated until death on the following day. The main clinical events of patient A are reported in Supplementary Table S1.

**Table 1:** Phenotypic susceptibility antibiotic testing analysis on *Acinetobacter baumannii* isolates from blood cultures.

		Acinetobacter_540_patient_A Acinetobacter_5577_patient_B				Acinetobacter_patient_A RS	
Drug class	Drug	MIC mg/L	EUCAST 2023 breakpoint	MIC mg/L	EUCAST 2023 breakpoint	MIC mg/L	EUCAST 2023 breakpoint
Aminoglycosides (systemic infections)	Amikacin	>16	*S* < =8; *R* > 8	>16	*S* < =8; *R* > 8	>16	*S* < =8; *R* > 8
	Gentamicin	>4	*S* < =4; *R* > 4	>4	*S* < =4; *R* > 4	>4	*S* < =4; *R* > 4
	Tobramycin	>4	*S* < =4; *R* > 4	>4	*S* < =4; *R* > 4	>4	*S* < =4; *R* > 4
Cephalosporins	Ceftriaxone	Resistant		Resistant		Resistant	
	Cefazolin	Resistant		Resistant		Resistant	
	Cephalexin	Resistant		Resistant		Resistant	
	Cefadroxil	Resistant		Resistant		Resistant	
	Cefiderocol	>128	Resistant	1	Sensitive	1	Sensitive
Fluoroquinolones	Ciprofloxacin	>1	*S* < =0.001; *R* > 1	>1	*S* < =0.001; *R* > 1	>1	*S* < =0.001; *R* > 1
	Levofloxacin	>2	*S* < =0.5; *R* > 1	>2	*S* < =0.5; *R* > 1	>2	*S* < =0.5; *R* > 1
Tetracyclines	Tetracycline	Resistant		Resistant		Resistant	
	Doxycycline	Resistant		Resistant		Resistant	
	Tigecycline^b^	0.5		0.5			
Carbapenems	Imipenem	>8	*S* < =2; *R* > 4	>8	*S* < =2; *R* > 4	>8	*S* < =2; *R* > 4
	Meropenem^b^	>16	*S* < =2; *R* > 8	>8	*S* < =2; *R* > 8	>8	*S* < =2; *R* > 8
Miscellaneous agents	Trimethoprim/sulfamethoxazole	>4/76	*S* < =2; *R* > 4	>4/76	*S* < =2; *R* > 4	>4/76	*S* < =2; *R* > 4
	Colistin^b^	1	*S* < =2; *R* > 2	1	*S* < =2; *R* > 2		*S* < =2; *R* > 2

b Minimal inhibiting concentration confirmed by epsilometer test (E-test).

Overview of antibiotic susceptibility testing results for *Acinetobacter baumannii* isolates: Patient A (*A. baumannii*_5406), Patient B (*A. baumannii*_5577), and Patient A rectal swab (RS). The data include drug classes, specific antibiotics, minimum inhibitory concentrations (MICs, mg/L), and the European Committee on Antimicrobial Susceptibility Testing (EUCAST) 2023 breakpoints. EUCAST 2023 Breakpoint column displays the EUCAST breakpoints for 2023, indicating susceptibility (S) or resistance (R) to the antibiotic based on the MIC value.

On the same days, a 50-year-old man (Patient B) with acute myeloid leukemia undergoing second-line treatment in the same ward tested positive for carbapenem-susceptible *A. baumannii* through PCR testing on bronchoalveolar lavage on D2. Initial broad-spectrum antibiotic therapy with standard dose of meropenem (1 g every 8 h) and vancomycin was escalated the next day to 2 g meropenem every 8 h via extended infusion. Despite this, fever persisted, and on D4, BCs turned positive, isolating a FDC-susceptible CRAB (*A. baumannii_5577*), whose antibiotic susceptibility was analogous to that of the rectal isolates colonizing patient A (*A. baumannii_*patient A RS) (Table 1).

### Whole-genome analysis reveals no evident differences between *Acinetobacter baumannii* isolates

The genomic assembly of whole genome sequencing (WGS) of isolates 5406 and 5577 produced two high-quality draft genomes (completeness: 99.59%, contamination: 0.96%). Genome-based taxonomic assignment of the assemblies of isolates 5406 and 5577 confirmed that they belong to *A. baumannii* based on their genetic closeness (>95% ANI distance) to a set of 2,646 genomes from the NCBI annotated as *A. baumannii*. Among these genomes, isolate_5406 was closest with assembly GCA_022468295 (99.9362%, BioSample ID: SAMN25131663) and isolate_5577 with assembly GCA_003335945 (99.9352%, BioSample ID: SAMN09667783). Multi-locus sequence typing (MLST) of isolates 5406 and 5577, based on the specific alleles for genes of the Pasteur (cpn60, fusA, gltA, pyrG, recA, rplB, and rpoB) and Oxford (gltA, gyrB, gdhB, recA, cpn60, gpi, and rpoD) loci schemes, revealed that they belong, respectively, to sequence types ST2 and ST369 (Supplementary Table S2).

Comparison of the two isolate assemblies using different methods for ANI estimation revealed a genetic similarity of at least >99.9653%, suggesting that isolates 5406 and 5577 belonged to the same strain (Morris et al., 2021; Page, 2019). In particular, whole-genome alignment showed that sequences assembled from isolate_5406 spanned 99.98% of the sequences assembled in isolate_5577, with an ANI of 99.999% (Supplementary Table S3).

### Genomic characterization of *Acinetobacter baumannii* resistant strain revealed a unique deletion in a novel homolog of TonB-dependent receptor genes

Given the high genetic similarity between the two isolates despite their variance in FDC susceptibility, we conducted a variant calling analysis between the FDC-resistant isolate_5406 and the non-FDC-resistant isolate_5577 to detect variations that might explain the phenotype.

Sequencing the reads of isolate_5406 was mapped to the assembled genome of the FDC-resistant isolate_5577, chosen as a reference, and was used to detect and annotate potential single nucleotide polymorphisms (SNPs) and insertion–deletion events (indels).

We reported only five significant genetic differences between the two isolates, with three falling into predicted CDS (Supplementary Table S3).

In particular, we detected a base substitution (G → A) in the CDS predicted to codify the ribonuclease I (locus tag: EECGJPIA_00294) and an insertion (A → AT) in a CDS that appear to codify for a protein that belongs to the isoprenylcysteine carboxylmethyltransferase family protein (locus tag: EECGJPIA_00493).

More interestingly, we detected a 10-base deletion (CGAGGCTATAA → C) inside a CDS identified by the annotation software Prokka as coding for a hypothetical protein (locus tag: EECGJPIA_03186). Sequence similarity search of the translated CDS using blastp in the nr/nt NCBI database resulted in a strong sequence homology with proteins annotated as TonB-dependent receptors (Table 2). In addition, from an amino acid homology search, this protein was found to share a high percentage identity (96.9%) with a large portion (714/1,057 aa) of a putative receptor protein member of the UniRef90 cluster R8Y1N4^8^, containing the Secretin/TonB short N-terminal domain.

**Table 2:** Variant calling.

	Isolate annotation							Variant calling of 5577 vs. 5406			
*A. baumannii*_5577 Contig ID	CDS type	CDS start position	CDS end position	strand	Locus tag	Prokka product annotation	Blastp majoritary annotation	Variant position	REF	ALT	INFO
gnl|X|EECGJPIA_2	gene	6370	7032	–	EECGJPIA_00294	Hypothetical proteins	Ribonuclease I	6861	G	A	SNP
gnl|X|EECGJPIA_2	gene	202652	203074	–	EECGJPIA_00493		Isoprenylcysteine carboxylmethyltransferase family protein	202679	A	AT	INS
gnl|X|EECGJPIA_22	gene	100	2247	–	EECGJPIA_03186		TonB dependent receptor	777	C	T	SNP
gnl|X|EECGJPIA_22	gene	100	2247	–	EECGJPIA_03186		TonB dependent receptor	126	CGAGGCTATAA	C	DEL

The table presents the results of variant calling contextualized to specific contigs and coding sequences (CDS) of isolate 5577, used as reference. The table includes details about the contig identity (reference to the genomic context of the analyzed CDS), CDS type, start and end positions of the CDS, strand orientation, CDS locus tag, protein product predicted by Prokka, blastp result, and information about detected genetic variations. The SNP position indicates the position of the genetic variation within the coding sequence. The reference (REF) and alternative (ALT) columns specify the genetic variation.

The column information provides additional information about the detected genetic variation, such as the type of variation (e.g., “ins” for insertion, “snp” for single nucleotide polymorphism, and “del” for deletion).

The examination of Conserved Domain Architecture revealed the protein’s shared homology with outer membrane receptors primarily associated with iron uptake and transport in bacterial superfamily proteins of TonB-dependent receptors.

TonB-dependent receptor family is a family of proteins found in the outer membrane of Gram-negative bacteria. These receptors are involved in the active transport of scarce resources such as iron, vitamin B12, and other nutrients into the cell, utilizing the energy provided by the TonB-ExbB-ExbD complex.

The deletion of isolate _5,406 modifies the transcript of a newly identified homolog of the TonB receptor. The deletion of 10-bases was confirmed by PCR sequencing of the FDC-resistant isolate_5406, compared to the FDC-susceptible isolate_5577 (Supplementary Figure 1).

The novelty of the association between this particular TonB deletion and phenotypic FDC resistance in a clinical isolate was assessed by a multiple sequence alignment of the TonB-dependent receptor genes of isolates 5577 and 5406 against 3,845 homologs in *A. baumannii* ST 2 genomes. The analysis confirmed that the 10-base deletion identified by our analysis was unique and specific to FDC-resistant isolate_5406.

### The amino acid deletion of the TonB-dependent receptor led to an alteration of the predicted 3D protein structure

**Figure 1:**
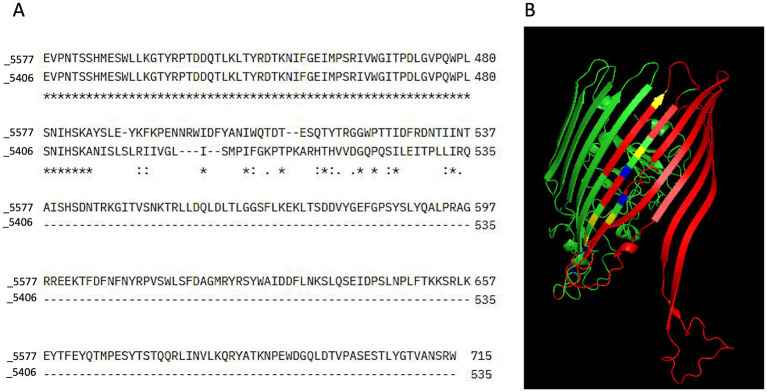
Sequence alignment and 3D structure prediction. (A) Sequence alignment was performed using Clustal Omega to compare the amino acid sequences of the sensitive (5577) and resistant (5406) bacterial strains. In the alignment output, stars (*) indicate positions where the amino acid residues are fully conserved between the two sequences. Colons (:) represent positions with strongly similar properties, reflecting conservative substitutions between residues with similar biochemical characteristics. Dots (.) denote weakly similar properties, indicating semi-conservative substitutions. Empty spaces indicate the absence of any similarity between the sequences at that position, while dashes (-) represent alignment gaps introduced to optimize sequence matching. **(B)** The 3D structure of the TonB-dependent receptor was predicted using Phyre2. In the resulting model, regions missing in the resistant strain (5406) were highlighted in red to indicate structural deletions. Residues representing conservative substitutions were shown in blue, while positions with complete conservation across all sequences were displayed in green. Semi-conserved substitutions were highlighted in yellow, representing residues with similar but not identical biochemical properties. The predicted structure was visualized and analyzed using PyMOL.

By aminoacidic sequence alignment of the novel homolog TonB-dependent receptor, we were able to demonstrate that the 10-base deletion detected in the FDC-resistant strain is predicted to cause a shift in the translational frame leading to a premature stop codon (Figure 1A).

In its native form, the predictive 3D protein structure of the receptor showed the presence of 6 alpha helices, one transmembrane helix, and 32 beta strands. In the deleted form, the amino acid residues spanning from position 547 to 714 were missing (Figure 1A) and, consequently, 6 beta strands and one alpha-helix were absent (Figure 1B). The predicted divergence in sequence composition between the resistant and sensitive strains therefore points toward a potential disruption in proper domain formation in the FDC-resistant strain.

### FDC uptake is inhibited in the clinical-resistant isolate harboring the novel 10-base deletion in TonB-dependent receptor

**Figure 2:**
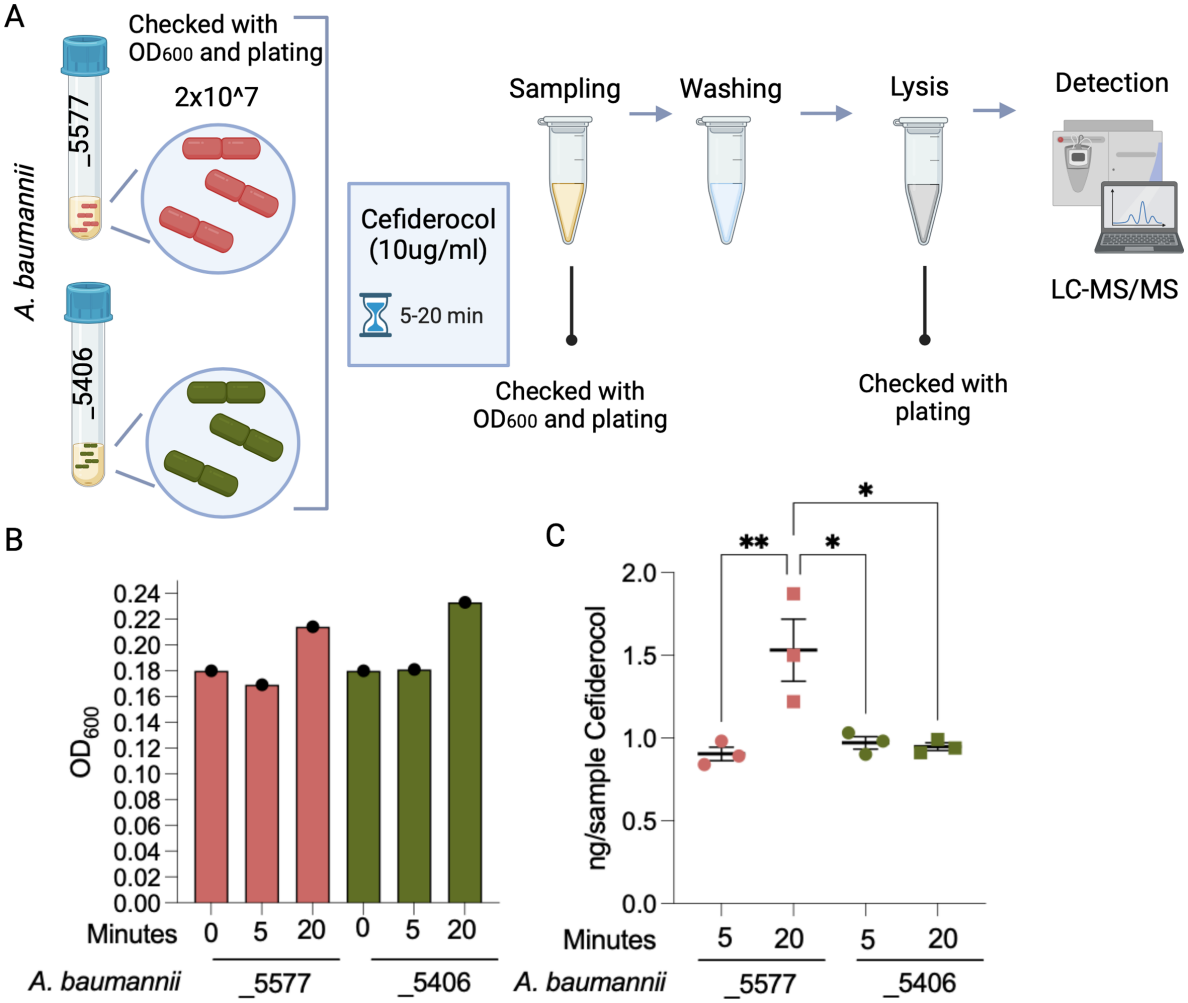
Experimental detection of FDC uptake in *A. baumannii* strains. **(A)** Experimental design for detecting FDC uptake in two *A. baumannii* strains: *A. baumannii*_5577 (susceptible) and *A. baumannii*_5406 (resistant). Bacterial cells were standardized to a concentration of 2×10^7 cells and exposed to an FDC concentration of 10 μg/mL. Cultures were incubated for 5 and 20 min. At each time point, a portion of the culture was sampled, subjected to washing, and then lysed to measure the FDC concentration within the bacterial cells. **(B)** Quantification of *A. baumannii* strains was reported as OD600 and measured before the FCS treatment and at 5 and 20 min during FDC treatment. **(C)** Graph showing the quantified amount of FDC detected using mass spectrometry within the lysed bacterial cells. The measurements are normalized based on the optical density (OD600) of the bacterial cells in the vial after the washing procedure. *p*-value <0.05 (*) and *p*-value <0.001 (**). Created with BioRender.com.

To assess a potential impairment mediated by the unfunctional novel homolog of the TonB-dependent receptor, the FDC uptake within the two bacterial isolate cells was tested. To study the role of the genetic mutation in the novel homolog of TBDRs and activate iron transport mechanisms, the experiment was conducted in an iron-depleted, cation-adjusted HB medium (ID-CAMHB). To avoid the activation of existing metal beta-lactamases, we excluded the addition of Zn2+. The number of bacterial cells was normalized and checked at 2×10^7 to have the same amount of cells for isolates_5406 and _5577. FDC was quantified by LC–MS/MS in washed bacterial isolate pellets grown in ID-CAMHB supplemented with 10 ug/mL of FDC and incubated for 5 and 20 min (Figure 2A). The antibiotic concentration was chosen based on preliminary tests, to prevent cell lysis and enable detection within the cells. The growth of the two isolates in the ID-CAMHB medium was monitored at 0, 5, and 20 min. OD600 measurements and plating (data not shown) indicated that both strains maintained cell viability and growth after 20 min of culturing (Figure 2B).

While no disparity in uptake was observed between total μg of FDC in bacterial cells of the isolates after 5 min of incubation, a noticeable enhancement in FDC uptake emerged exclusively in the susceptible *A. baumannii*_5577 strain after a 20-min incubation period while being absent in the resistant *A. baumannii_5406* strain.

This analysis unveiled the substantial capacity of *A. baumannii*_5406-resistant strain to inhibit the intracellular accumulation of FDC upon mutation of the novel homolog of TonB-dependent receptor (Figure 2C), thereby justifying the phenotypic FDC resistance and treatment outcome observed in clinical practice.

## Discussion

In this study, we report the identification, genomic characterization, and functional analysis of a new mutation causing the loss of functionality of a previously undescribed TonB-dependent receptor analog, associated with *in vitro* and *in vivo* phenotypic resistance to the new siderophore cephalosporin FDC in *A. baumannii*. By conducting this study, we have identified an analytical research workflow capable of supporting the definition of new genetic markers both from a genomic and functional point of view, trying to set up an analysis modality that can favor the identification of markers actually useful for the implementation in clinical practice.

The integration of variant calling-based whole-genome sequencing and LC/MS–MS techniques can provide a more comprehensive understanding of resistance mechanisms, potentially complementing routine clinical screening to inform therapeutic strategies and improve epidemiological monitoring. Indeed, while numerous Gram-negative bacteria exhibit *in vitro* susceptibility to FDC, the *in vivo* development of acquired resistance to this antibiotic, particularly in *A. baumannii*, remains poorly investigated, with limited studies addressing the mechanisms beyond mutations in *β*-lactamases or penicillin-binding proteins and TonB receptors (Nordmann et al., 2022).

In virtue of the established understanding that the main mechanism of FDC resistance in various pathogens involves the iron uptake system and iron-dependent challenges in performing FDC MIC microdilutions, it remains crucial to establish complementary techniques to rapidly detect novel *in vivo* mutations and assess the functional capacity of bacteria to import and accumulate FDC. The integration of variant calling-based WGS analysis and LC/MS–MS into isolates’ characterization could enforce the clinical practice for the monitoring of *in vivo* resistance and provide an epidemiological overview of bacterial strains and resistance mechanisms.

Owing to its recent approval, FDC resistance in the clinic is still sporadic, but it can still emerge. Our patient A was initially colonized by an FDC-susceptible *A. baumannii* that probably evolved in the FDC-resistant *A. baumannii*_5406 after 6 days of FDC treatment. A significant limitation of this study is the unavailability of the initial colonizing isolate that would have proven (with a higher degree of certainty) the development of resistance under drug pressure rather than the *de novo* acquisition of a resistant strain from the hospital environment. Concordant with our hypothesis, our analysis shows the high similarity between the FDC-resistant *A. baumannii*_5406 sequence from patient A and the FDC-susceptible *A. baumannii*_5577 strain, isolated on the same days in patient B, admitted to the same ward. Our findings sustain the hypothesis of a colonization of both patients by the same *A. baumannii* strain that became FDC-resistant in patient A following FDC treatment.

Notably, the initial bioinformatic analysis of WGS revealed no genotypic differences between the FDC-resistant and the FDC-susceptible strains. This finding did not align with the clinical evidence based on MIC analysis and prompted us to deeper evaluations. As WGS is increasingly used in clinical practice, our experience strongly supports the need for constant collaboration between bioinformatics and microbiologists in the interpretation of genomic data, so as to avoid the loss of important information.

In our case, an additional variant calling analysis was performed and led to the identification of a promising, newly described 10-base deletion in a CDS (locus tag: EECGJPIA_03186), which was found to code for a homolog of TonB-dependent receptor. During analysis, this particular mutation has never been observed in any *A. baumannii* isolate sequenced (and available in public databases), a situation compatible with the recent approval of FDC.

The TonB-dependent receptor complex plays a pivotal role in nutrient uptake, particularly the transport of iron across the outer membrane of bacterial cells. Previous studies have shown that mutations in genes encoding iron transport proteins, such as TonB and other transporters, can lead to reduced uptake of antibiotics, thereby promoting antibiotic resistance (Padovani et al., 2023; Teran et al., 2024; Egge et al., 2024; Moynié et al., 2017). Alterations or disruptions in this transport system can have significant consequences for nutrient availability and, consequently, antibiotic susceptibility in *both A. baumannii* and *P. aeruginosa* (Padovani et al., 2023; Teran et al., 2024; Egge et al., 2024; Moynié et al., 2017).

In our study, 3D protein modeling allowed us to demonstrate that the 10-bases deletion found in our FDC-resistant clinical strain was able to disrupt the secondary protein structure, leading to alterations compatible with a loss of the protein function as a transporter, potentially contributing to its resistance phenotype.

This hypothesis was then demonstrated by functional experiments using a depleted medium for iron, which resembles the limited availability of free iron *in vivo*, and LC/MS–MS-based detection of intracellular FDC demonstrated reduced FDC uptake in the resistant strain.

Overall, this study provides valuable insights into the variant calling analysis, necessary to highlight *in vivo* genetic adaptations that *A. baumannii* can undergo to develop antibiotic resistance. The identification of a novel variant of *A. baumannii* resistant to FDC and mutated in a coding sequence for a homolog of TonB receptors confirms the involvement of this entrance mechanism in the development of FDC resistance. The LC/MS–MS analysis was relevant for a functional assessment, testing the accumulation of FDC within bacterial cells. While the integration of advanced methodologies, such as variant calling-based whole-genome sequencing and LC/MS–MS, could potentially allow for the early identification of resistance patterns and improve therapeutic strategies, their routine use in clinical diagnostics may be limited currently by factors such as the availability of specialized equipment, the labor-intensive nature of preparing ID-CAMHB, and the need for trained personnel. Further studies and feasibility plans incorporating variant calling analysis of WGS and functional mass spectrometry into clinical practice could help address challenges in FDC-resistant screening and improve epidemiological monitoring.

## Data Availability

The datasets presented in this study can be found in online repositories. The names of the repository/repositories and accession number(s) can be found in the article/Supplementary material.

## Footnotes

^1^ https://www.fetroja.com/

^2^ https://www.eucast.org/ast-of-bacteria/warnings

^3^ http://www.eucast.org

^4^ https://github.com/FelixKrueger/TrimGalore

^5^ https://github.com/tseemann/snippy

^6^ https://www.iss.it/documents/5430402/5748333/eurl_4_guidelines_cluster_analysis_wg_ngs_v-1.pdf/50cb9fbe-4504-789a-01a3-93e55d52cd0c?t=1623330424004

^7^ http://www.pymol.org/pymol

^8^ Q6FC78
