# Supplementary material for: Distinct chromosomal mutation associated with cefiderocol resistance in Acinetobacter baumannii: a combined bioinformatics and mass spectrometry approach to unveil and validate the in vivo-acquired chemoresistance
Source: Front Microbiol. 2024 Dec 18;15:1480322. doi: 10.3389/fmicb.2024.1480322 (PMC11688269; doi:10.3389/fmicb.2024.1480322)
Supplement: Supplementary file 1 [file Data_Sheet_1.docx]

**Supplementary Material**

WGS_5577 GTATTACGCCTGACTTGGGTGTTCCGCAGTGGCCTTTAAGCAATATCCACTCTAAAGCTT

PCR_5577 GTATTACGCCTGACTTGGGTGTTCCGCAGTGGCCTTTAAGCAATATCCACTCTAAAGCTT

PCR_5406 GTATTACGCCTGACTTGGGTGTTCCGCAGTGGCCTTTAAGCAATATCCACTCTAAAG---

*********************************************************

WGS_5577 ATAGCCTCGAATATAAGTTTAAGCCTGAGAATAATCGTTGGATTGATTTCTATGCCAATA

PCR_5577 ATAGCCTCGAATATAAGTTTAAGCCTGAGAATAATCGTTGGATTGATTTCTATGCCAATA

PCR _5406 -------CGAATATAAGTTTAAGCCTGAGAATAATCGTTGGATTGATTTCTATGCCAATA

*****************************************************

WGS_5577 TTTGGCAAACCGACACCGAAAGCCAGACATACACACGTGGTGGATGGCCAACCACAATCG

PCR_5577 TTTGGCAAACCGACACCGAAAGCCAGACATACACACGTGGTGGATGGCCAACCACAATCG

PCR_5406 TTTGGCAAACCGACACCGAAAGCCAGACATACACACGTGGTGGATGGCCAACCACAATCG

************************************************************

WGS_5577 ATTTTAGAGATAACACCATTATTAATACGGCAATAAGCCATTCTGACAATACGCGTAAAG

PCR_5577 ATTTTAGAGATAACACCATTATTAATACGGCAATAAGCCATTCTGACAATACGCGTAAAG

PRC_5406 ATTTTAGAGATAACACCATTATTAATACGGCAATAAGCCATTCTGACAATACGCGTAAAG

************************************************************

WGS_5577 GCATTACCGTCAGCAATAAAA

PCR_5577 GCATTACCGTCAGCAATAAAA

PCR_5406 GCATTACCGTCAGCAATAAAA

*********************

**Supplementary Figure_1: DNA based alignment between two isolates**

Clustal W alignment of PCR sequencing conducted using FW and Rev primers to validate the 10-base deletion in the resistant mutant strain. The deletion was confirmed, as shown by the Clustal W alignment. Asterisks indicate perfect matches, while dashes indicate the sequence deletion. WGS_5577 represents the sequence derived from the WGS approach of the sensitive isolate.

**Supplementary tables:**

The supplementary Table_2 is available to <https://docs.google.com/spreadsheets/d/11RHe0Y66nhstr-jVerAfGWZNjqqrUYt0k3aSSXGV550/edit#gid=1494938383>


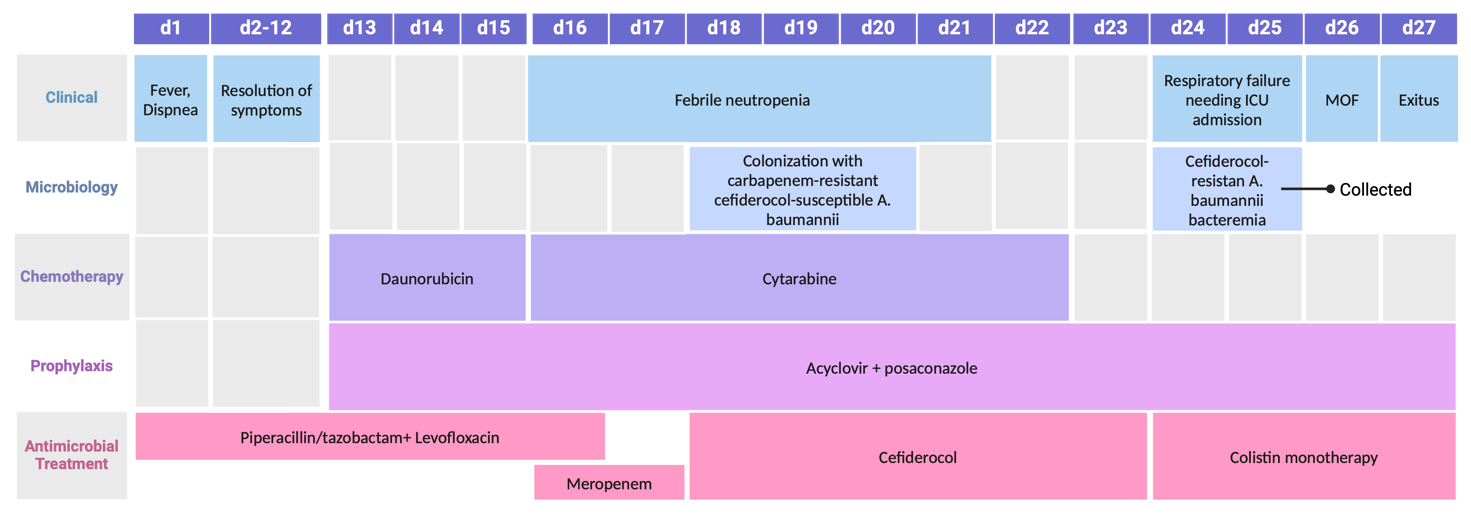


**Supplementary Table 1:** Treatment and microbiological timeline for Patient A.

**
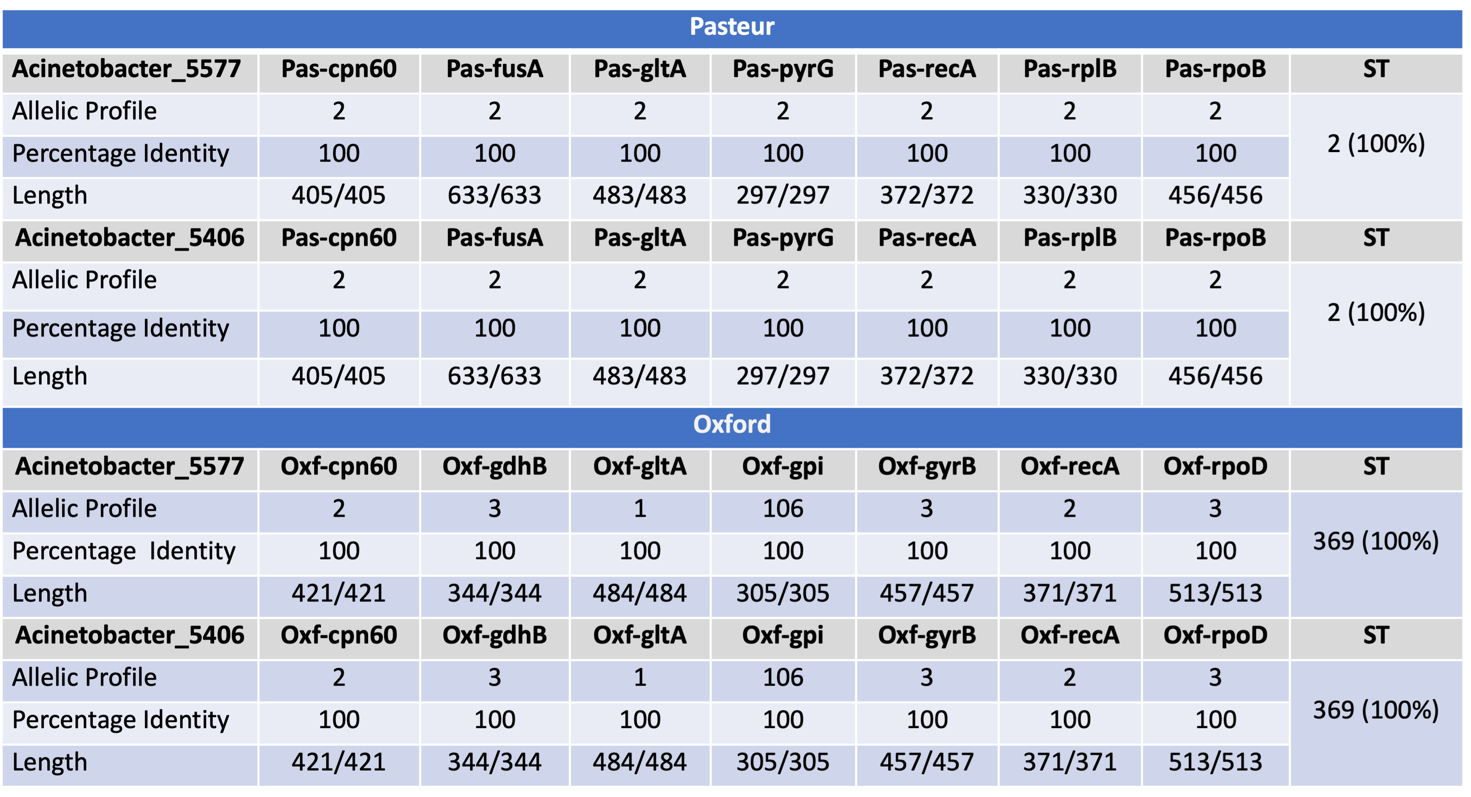
**

**Supplementary Table 3:** Multi-locus sequence typing of *A. baumanni* assemblies

Multi-locus Sequence Typing (MLST) results for two *Acinetobacter baumannii* isolates, _5577 and _5406, using both Pasteur and Oxford database which uses different sets of housekeeping genes for sequence analysis. The table displays allelic profiles, percentage identity, and length information for each of the analyzed loci. Sequence Type (ST) represents a unique combination of allelic variants across the selected housekeeping genes. It is a numerical identifier assigned to each isolate based on its allelic profile. Percentage Identity shows the percentage identity between the analyzed isolate and the reference strain for each gene locus in the MLST scheme. The length column provides the number of nucleotides in the sequence alignment for each gene locus.
